# Monthly Summary

**Published:** 1892-01

**Authors:** 


### Monthly Summary.

Clinical Nights at the Societies.—"That all things change while nothing perishes," is a saying as old as the Roman hills. It is, perhaps, a tremendous and crushing thought for some of us, that our "things of no worth" go ricochetting over the universe, while seasons come and go, and yet so it is. But, while the scenes and sounds, say of Cicero's Rome, are again enacted and heard in this London of to-day, a modern Horace, telling of scheming mammas and foreseeing daughters, as of old by Tibur's banks, a modern Livy narrating of storms by sea and portents by land, all very much as of old, save perhaps the manner of men and the cloaking of ideas in modern guise of words. The gist of the matter is, as was said, that while all things change, the things still persist. Were it possible to know the bearings of the age in which one lives, we should say to-day was the epoch of practicality. Formerly, the grass to which we ephemeral men are likened was allowed to fade decently and lingeringly through the genial action of the sunshine; but, alas, to-day it is cut and dessicated and generally done for by machinery, and so its day is shortened: hence, life to us is no longer a procession of time, it has become a rush of moments. Admitting the breathless haste in which Nineteenth Century mortals live and move, and have their being, we become impressed with a vague longing to make the most of what goods the gods send us, we eschew the sober picking of bones and fly to meat extracts and concentrated essences. The material and the mental sides of our nature are certainly not at variance in this matter, and so it happens that while our physical digestion craves for a diet of little and often, so does our intellectual appetite assimilate a pabulum wherein brief, pithy statement of facts replaces excursuses, massive in form and bristling in erudition. Certainly one of the greatest improvements of these latter-day things has been the substitution of short clinical histories for the older-fashioned academical papers in which our forefathers revelled. Of course, formal papers have their own value and serve a very useful purpose, but for the requirements of practical men, such as dentists are, such evenings as the one which the Odontological Society devoted at its last meeting, to "Casual Communications," have great attractions. It is given but to few men to compose an elaborate paper, there are, however, few who cannot stand up for ten minutes and describe some case of uncommon interest or some time-saving device, so that the burden of supplying material is less on casual nights. Perhaps it would be hard to find a better end-up for an interesting practical evening than that which Mr. Hooten contributed at the meeting in question. A reference to our "report" will show how admirably practical and to the point Mr. Hooten's remarks were. As "one of the crowd" it is not for us to offer advice, but we may, perhaps, offer our congratulations to the secretaries for the excellent programme of the meeting, and even express a hope that there may be several more such evenings in store for us.—*Dental Record.*

---

PYOKTANIN AS AN ANTISEPTIC.—Whenever the conservative dentist or physician sees a remedy unduly vaunted and its virtues extravagantly extolled, he should be extremely careful in his first experiments with it. The chances are ten to one that it will be a disappointment. Many remember what absurd laudations were paid to pyoktanin when it was first heralded to the public. It was the "Pus Killer," and all other antiseptics might be retired at once. We tried the miserable nasty stuff once or twice, and that was sufficient. We discovered that it was a proprietary remedy, that the manufacturers were shrewdly making practitioners do the advertising, and from this they were coining money at the extortionate price at which it was sold.

Dr. Roswell Park, Professor of Surgery in the University of Buffalo, Medical Department, in *Annals of Surgery*, gives his experience with it. He says:

"It cannot be relied upon in cases of surgery except in a strength that is dangerous. As an injection in gonorrhœa, I

have had no experience with it, but find that the most of those who have tried it have met with disappointment. Upon granulating surfaces it does appear to be stimulating and to exert a desirable effect, but no more so than other substances within easy reach, and its stain is often undesirable. In ophthalmological practice it appears also to have scarcely come up to the requirements of the day. On the whole, then, it has but few qualities by which we are to commend it above numerous other drugs of its general class, while in all that may answer to the more scrupulous demands of aseptic surgery, it has proved in my hands—as in those of others who have tested it from the purely clinical standpoint—disappointing."

Exit pyoktanin in a blue-red cloud, leaving behind it but a sickening stench and an ineradicable stain.—*Dental Pract. and Advertiser.*

---

ERUPTION OF THE DECIDUOUS TEETH, AND GENERAL SYMPTOMS.—H. Augustus Aldred, D. D. S., Philadelphia, in *Arch. of Pœdriatics* (for Oct ), writes :

At about the fifth month after birth the process known as the eruption of the teeth begins; a double process, consisting of the gradual elongation and rising of the teeth, and the coincident absorption of the hard and soft tissues overlying them.

The alveolar borders are the first to show signs of the absorptive process, by a dissolution or melting of their approximated edges, thus gradually making a wider space for the advancing teeth. These rising in their sockets, the roots meanwhile lengthening, press upon the overlying gums, which, becoming thinner and thinner, finally allow the escape of the imprisoned teeth.

The symptoms of pathological dentition are loss of appetite, peevish fretfulness, wakefulness, feverish thirst, continuous suffering, bowels loose or constipated, tendency towards congestion of brain, and it may even terminate in death. The usual local signs of abnormal dentition are redness and swelling, followed by whiteness of gums, decided flow of saliva ("drooling"), desire to suck thumb or fingers, biting the ring or spoon with determination, alternately taking and refusing the breast, and desiring upright position (to counteract flow of blood).

The rule is that the lower teeth precede the upper of the same class two or three months, but not infrequently the upper precede the lower by the same difference in time. Again, the rule is that the teeth are erupted in pairs, with an interval between the different pairs, but occasionally a single tooth will appear a considerable time before its fellow, and in other cases two or three pairs will erupt coincidentally.—*Maryland Medical Journal.*

---

DEATH FROM TOOTH SEPSIS.—Dr. Miller informs us of a recent case of death succeeding a dental operation in Berlin, which illustrates the importance of the antiseptic precautions that he recommends in his writings. The Baroness Martha Von ——, engaged to be married to an officer of high rank in the German service, had a tooth filled. (Report does not say which tooth it was, but probably it was a lower molar). Soon after the operation peticementitis supervended, followed by great swelling of the lower part of the face and the neck. Laryngotomy was performed by Professor Schrotter, and every means employed to prevent the spread of the infection, but in vain. The young lady succumbed to the progress of the septicæmic condition, and died in fearful agony.—*Dental Pract. and Advertiser.*

---

A Russian writer recommends the flowers of the red rose as a remedy for the treatment of chronic diarrhœa. It is a favorite house remedy for this trouble among the Russian people. A handful of the dried flowers is made into an infusion. Children up to 5 years of age take a tumbler of the infusion in the course of a day. Adults may take from two to three tumblerfuls. Its taste is agreeable, especially if some sugar be added. It has cured some obstinate cases of diarrhœa in less than a week.—*Md. Med. Journal.*

TRIGEMINAL NEURALGIA AND IODIDE OF POTASSIUM.—In the last number of the *Neuroligisches Centralblatt*, reference is made to some singular facts related by Dr. Ehrmann as to the occurrence of severe facial neuralgia after the administration of even small doses of iodide of potassium. In the first case mentioned, a strong workingman of thirty-five suffered most intense pain in the forehead and teeth, with sensitiveness over the whole distribution of the fifth nerve, after taking fifteen grains of the drug. A second patient, after taking fifteen grains, had much pain in the region of the upper jaw, with pain and a tenderness in separate branches of the nerve, and also œdema of the eyelids on the left side. A third and a fourth patient also suffered similar symptoms after similar doses. They were associated in all cases with lachrymation and injection of the conjunctiva, but the symptoms rapidly vanished, and did not re-appear on a further administration of the drug. The cases are not only interesting, but important, for it is desirable to know as much as possible regarding any peculiar effects likely to be produced by a drug which is so frequently administered as an iodide of potassium.—*Dental Pract. and Advertiser.*

---

DARK SPOTS ON THE TEETH.—The next time you have such a case, try Dr. McCandless' plan, viz: add a drop or two of aromatic sulphuric acid to your paste of pumice and water, and continue using the soft rubber disk loaded with this mixture, and this stain disappears as if by magic. Floss silk charged with this preparation is a simple yet effective means of removing stains from between the teeth.—*Southern Dental Journal.*

---

A new society, to be known as the Western Association of Obstetricians and Gynæcologists, will come into existence soon. Geographically, its territory will include the Missouri river, cities of Iowa and Missouri, and the states and territories of Kansas, Nebraska, Colorado, New Mexico, Indian Territory and Oklahoma.—*Md. Medical Journal.*

THIRTY-SIX TEETH IN A SET.—*By George McDonald, L. D. S., Carleton Place, Ont.*—I read an item in the November "Items of Interest," written by J. W. Greene, P. D., Chillicothe, Mo., stating he knows a man who has *thirty-six* natural teeth and perfect in form. Some time ago I met a similar case at Bryson, Que. A gentleman called one morning to have a tooth removed which was annoying him on account of being loose, and, on examination, I found that he had *thirty-six* teeth, just as perfect in form and arch arrangement as any set of *thirty two* I have ever seen. The surplus numbers consisted of molars, the four being as perfectly formed as the others, and about the average size of wisdom teeth. It was one of those he wanted removed, the left superior. After the operation I examined it and found it free from caries; also found the remaining three free from caries. At present he lives at Portage du Fort, Que. Was not aware he had *thirty-six* teeth until I told him. First and only case I have ever seen.—*Dominion Dental Journal.*

---

A SAFE LOCAL ANÆSTHETIC.—Dr. T. C. Meaker, of Carbondale, Pa., reports in the "Items of Iterest," that a few drops of a five per cent. solution of carbolic acid in water, injected under the gum on each side of a tooth to be extracted, makes an excellent and safe local anæsthetic. Its effects, he claims, is almost instantaneous, and is particularly marked when there is swelling and inflammation about the tooth. As only one drop of carbolic acid is contained in twenty drops of the solution, the quantity used is too small to cause any constitutional disturbance.—*Dental Record.*

---

CLEANSING OF THE HANDS.—Carbolic acid is removed from the hands by bathing them for a sufficient time in alcohol and then anointing them with lanolin or vaseline. After the use of corrosive sublimate solution the hands should be bathed in a solution of common salt, 1 to 50, then washed with soap and water, and finally rubbed with landolin.—*Pharmac. Central.*

TEMPORARY SETS.—In inserting temporary sets immediately after extraction, it is usually thought advisable to have the anterior teeth project upwards about a third of their length into the sockets of the former teeth. In fitting these, however, it is sometimss difficult to declde just where to cut into the cast, as the loose edges of the gums turn inward and obscure the outline of the alveolus while the impression is being taken. To overcome this, Dr. Driscoll, in the October "Items," advises the use of rolls of soft wax inserted into the sockets in such a way as to come away with the expression. These are then trimmed to the depth the teeth are intended to go, the result being that the teeth, if filled to the model, will go to place in the mouth without infringing upon either the gum or the alveolus.—*Dominion Dental Journal.*

---

THE HUMAN BREATH —Prof. Brown Sequard made experiments to determine whether the human breath was capable of producing any poisonous effects. From the condensed watery vapor of the expired air he obtained a poisonous liquid. which, when injected under the skin of rabbits, produced almost immediate death. He ascertained that this poison was an alkalcid, and not a microbe. The rabbits thus injected died without convlusions, the heart and large blood-vessels being engorged with blood. Brown-Sequard considers it fully proved that the expired air, both of man and animals, contains a valatile poisonous principle which is much more deleterious than carbolic acid.—*Southern Dental Journal.*

---

OBTAINING THE BITE.—Dr. W. Goodfellow, of Sussex, New Brunswick, writes us that a process which never fails to secure a correct bite in articulating artificial teeth, is to direct the patient to open the mouth and then to see that the tongue is placed firmly against the roof of the mouth, *well back*, and held there while closing it. In this position it is impossible to advance the lower jaw.—*Dental Advertiser.*

MORTALITY OF THE HUMAN RACE.—The annual mortality on the whole earth may be estimated on the ground of recent statistical results at about 33,000,000 of persons, which constitutes an average 91,554 deaths per day, 3,730 per hour, and consequently sixty-two per minute. The average length of human life is thirty-eight years. One-fourth of the human race dies before attaining the seventh year; one-half defore attaining the sixteenth year, or in the course of this year. But one person among 10,000 attains the age of 100 years, while one among 500 attains ninety; one among 100 attains sixty. Married people live longer than the unmarried. Among 1,000 persons who have reached the age of seventy years, forty-three belong to the clergy or the political class, forty to the agricultural class, thirty-three are workmen, thirty-two are soldiers, twenty-nine are lawyers or engineers, twenty-seven are professors, twenty-four physicians.—*Internationaler Phar. General-Anzeiger.*

---

An interesting reproduction of Benjamin Franklin's historical experiment with the kite, under somewhat different conditions, has been carried out at the Blue Hill Observatory by Alexander McAdie. What Mr. McAdie has demonstrated is that electricity can be drawn from a kite high in the air in a cloudless sky. The kite discharged sparks from the lower end of an insulated wire reaching down to the earth, where an electrometer partly measured the increasing electric force. So nearly did the quantity of electricity in the upper air correspond to the height of the kite above the earth that the experimenter could usually determine whether the kite was rising or falling by simply looking at the needle of the electrometer.—*American Druggist.*

---

PROFUSE HEMORRHAGE.—Never give stimulants in a case of profuse hemorrhage. The faint feeling, or irresistible inclination to lie down, is Nature's own method of circumventing the danger, by quieting the circulation and lessening the expulsive force of the heart, thus favoring the formation of clot at the site of injury.—*Clinique.*

